# Efficacy of quadratus lumborum block in the treatment of acute and chronic pain after cesarean section: A systematic review and meta-analysis based on randomized controlled trials

**DOI:** 10.1097/MD.0000000000036652

**Published:** 2024-01-26

**Authors:** Honghong Du, Xiuqin Luo, Min Chen, Siren Shi, Jianyong Zhao

**Affiliations:** a Department of Anesthesiology, Linping Campus, The Second Affiliated Hospital of Zhejiang University School of Medicine, Hangzhou, China; b Departments of Neonatology, Linping Campus, The Second Affiliated Hospital of Zhejiang University School of Medicine, Hangzhou, China.

**Keywords:** analgesia, cesarean section, meta-analysis, quadratus lumborum block

## Abstract

**Background:**

This analysis aimed to explore the analgesic effects of quadratus lumborum block on acute and chronic postoperative pain among patients undergoing cesarean section.

**Methods:**

PubMed, Cochrane, Embase, Web of Science, China National Knowledge Infrastructure, Wanfang, and VIP databases for Randomized Controlled Trials (RCTs) that focused on the use of quadratus lumborum block in cesarean section procedures were searched from the inception of the databases until December 2022. Studies were screened based on inclusion and exclusion criteria, and were then conducted for quality assessment and data extraction. Meta-analysis was performed using Stata 15.0 software. Two researchers independently screened the studies, extracted data, and evaluated the risk of bias for the included studies. In case of any disagreements, it was resolved by consultation with a third party opinion.

**Results:**

A total of 21 studies involving 1976 patients were finally included, with an overall acceptable study quality level. Compared to the control group, the administration of Quadratus Lumborum Block (QLB) resulted in significant reduction in the postoperative 24-hour visual analog scale (VAS) score (WMD = −0.69, 95% CI: −1.03 ~ −0.35, *P* < .001) and the consumption of opioid analgesics within 24 hours after surgery (WMD = −2.04, 95% CI: −2.15 ~ −1.92, *P* = .002). The incidence of chronic pain 3 months QLB (OR = 0.41, 95% CI: 0.09 ~ 1.88, *P* = .253) and 6 months (OR = 0.83, 95% CI: 0.33 ~ 2.07, *P* = .686) after surgery were observed to increase as compared with the control group.

**Conclusions:**

The use of QLB for postoperative analgesia after cesarean section, particularly in the relief of acute postoperative pain, had been proven to significantly decrease the VAS score and morphine consumption within the first 24 hours after surgery. However, further studies are needed to determine its impact on managing chronic postoperative pain.

## 1. Introduction

The global incidence of cesarean section (CS) surgery is increasing, particularly in low-income and middle-income countries.^[1]^ According to the assessment of CS trends from World Health Organization (WHO) in 21 countries, the overall rate increased from 26.4% to 31.2%.^[2]^ In China, the rate of deliveries by CS was 29% in 2008, which increased to 35% by 2014. However, a recent study indicated that the increase in CS in China had been more gradual compared to other countries, which accounted for a significant 41.1% of all deliveries between 2012 and 2016.^[3]^ Currently, most cesarean sections are performed under anesthesia using spinal cord or epidural techniques. While spinal anesthesia provides excellent postoperative analgesia after CS surgery, its effects typically last only a few hours, leaving patients vulnerable to severe pain afterward.^[4]^ Evidence indicated an association between postoperative pain after CS surgery and the risk of postpartum depression.^[5]^ Mothers experiencing severe postoperative pain had a higher risk of post-natal depression, which could have negative effects on the development of their children.^[6]^ These findings highlight the need for more effective strategies for managing postoperative pain.

With the incorporation of ultrasound in the regional block, it has been discovered that the trunk block is highly effective in alleviating pain after surgery and reducing the necessity for opioids.

This approach aims to minimize maternal opioid intake, thereby reducing the exposure of newborns to opioids through breastfeeding.^[7]^ The quadratus lumborum block (QLB) can allow the medication spread more extensively from the transverse abdominal muscle plane to the paravertebral space. This can relieve visceral and somatic pain, and its usage for postoperative analgesia is becoming more prevalent.^[8]^ Over the past few years, numerous randomized controlled trials (RCTs) had been conducted to investigate the role of QLB in post-cesarean section analgesia. However, the findings regarding the effectiveness of QLB in relieving postoperative acute and chronic pain had been inconsistent.

This systematic review and meta-analysis provided a comprehensive analysis of current existing prospective randomized controlled trials (RCTs) to assess the effectiveness of QLB analgesia techniques in managing acute and chronic pain after cesarean section surgery. The primary endpoints were postoperative 24-hour visual analog scale (VAS) scores, the consumption of opioid drugs within 24 hours after surgery, the occurrence rate of chronic pain after 3 months, and the occurrence rate of chronic pain after 6 months. This study could improve the clinical patient prognosis, accelerate patient recovery, and provide guidance for implementing multimodal analgesia schemes in clinical practice.

## 2. Methods

### 2.1. Retrieval strategy

Studies on the association between cesarean section surgery, quadratus lumborum block, and randomized controlled trials reported in PubMed, Cochrane, Embase, Web of Science, China National Knowledge Infrastructure, Wanfang, and VIP databases were searched from the inception of the databases until December 2022. Subject headings and free words were used. The search formula used was: (Cesarean section OR C-section OR CS) AND (quadratus lumborum block OR quadratus lumbois block OR QLB) AND (randomized controlled trial OR RCTs). We excluded irrelevant studies by screening the titles and abstracts. We further filtered related articles by reading the full text, according to inclusion and exclusion criteria. Additionally, we manually searched the references in the selected literature.

### 2.2. Inclusion and exclusion criteria

The inclusion criteria for this meta-analysis were as follows: Patients undergoing cesarean section; RCTs; Exposure factor: Implementation of QLB before or after surgery; Outcome measures: Score of VAS at 24 hours after operation, consumption of opioids at 24 hours after operation, incidence of chronic postoperative pain after 3 months and incidence of chronic postoperative pain after 6 months. The following criteria were considered for exclusion: Conference abstracts; Full text was not available or data was incomplete; Studies without sufficient data; Unadjusted confounding factors; Duplicated publications; Theses or dissertations.

### 2.3. Data extraction and literature quality evaluation

The retrieved records were imported into the EndNote20 document management software for de-duplication. Two researchers independently reviewed the title and abstract and excluded the studies that did not meet the inclusion criteria. In case of any discrepancies during the screening process, a thorough review and discussion of the documents were undertaken until a consensus was reached. If differences persisted, either consultation was sought or a third party opinion was obtained. The following information from each study was extracted: first author, year of publication, sample size, patient age, drug and dose, a score of VAS at 24 hours after operation, consumption of opioids at 24 hours after operation, incidence of chronic postoperative pain after 3 months and incidence of chronic postoperative pain after 6 months. To evaluate the risk of bias in the included studies, 2 researchers independently used the bias risk assessment tool for RCT in the Cochrane evaluation system.^[9]^ The evaluation was conducted using 7 aspects: the generation of random sequences, allocation concealment, blinding of implementers and researchers, blinding of outcome assessment, integrity of result data, selective reporting, and other biases. In case of any disagreement during the evaluation process, it was resolved either through consultation or by seeking a third-party opinion.

### 2.4. Statistical indicators

The Cochrane bias assessment risk was conducted using Revman 5.4 software. Meta-analysis was performed using Stata 15.0 software. The heterogeneity of the included studies was evaluated using the chi-square test. If the *P* value ≥ .1 and I² statistic < 50%, the heterogeneity was considered to be not high, and the fixed-effect model was used to calculate the combined effect. Conversely, if the I^2^ statistic ≥ 50%, indicating significant heterogeneity between studies, the random-effect model was used and a forest plot was generated. Sensitivity analysis was conducted to assess the stability of the results by observing the change in the overall effect by removing each study to ensure the robustness of findings. A funnel plot was used to assess the publication bias for included studies. All statistical tests were bilateral, and a *P* value < .05 was considered statistically significant.

## 3. Results

### 3.1. Baseline characteristics and quality evaluation of included studies

**Figure 1.**
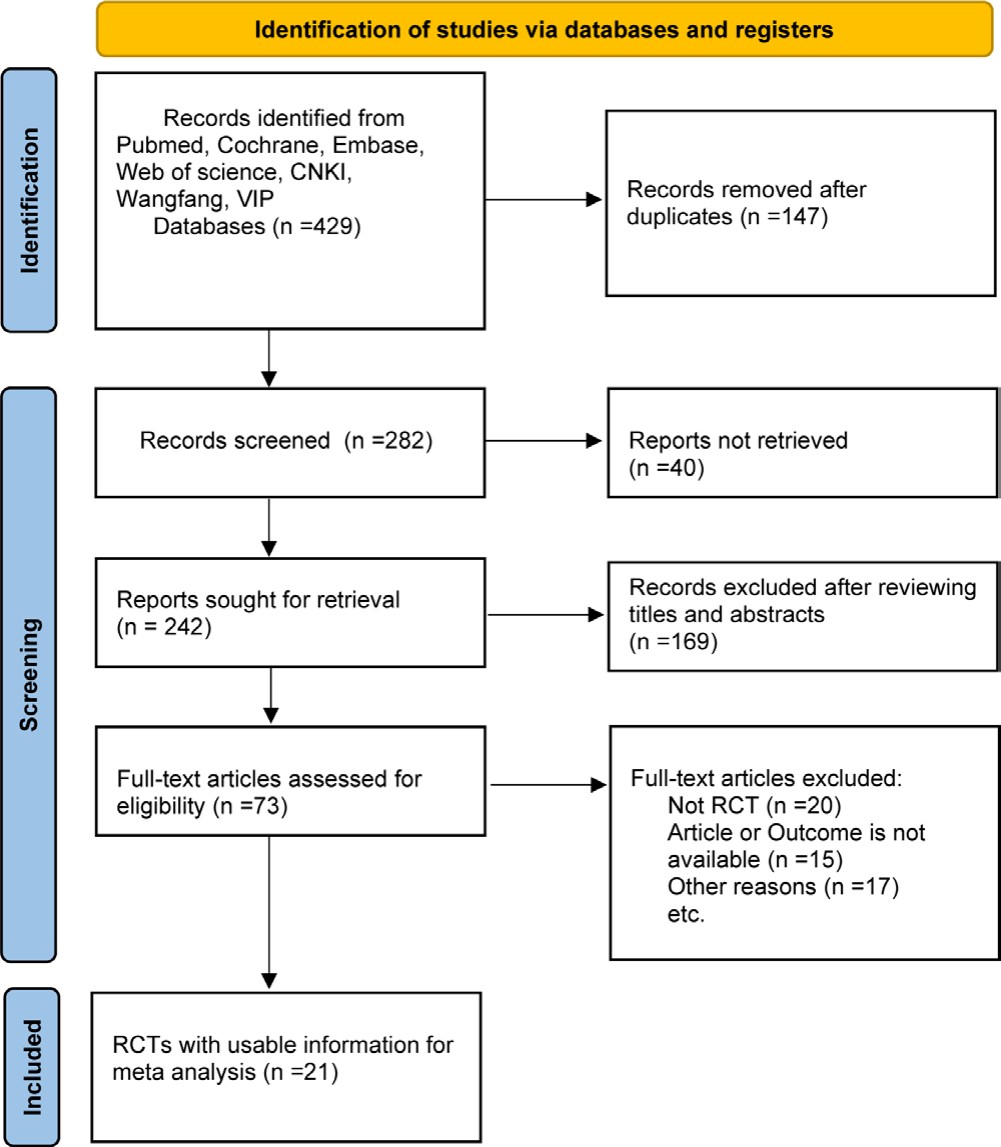
Flow chart of included literature screening.

A total of 429 studies were retrieved, 147 studies were excluded after repeated screening, and 40 studies were not retrieved. After 169 studies were excluded based on title and abstract reading, 73 studies were included for the full-text screening. Finally, a total of 21 studies were included for meta-analysis, as shown in Figure 1.

In the literature review, a total of 21 studies were included with 1976 patients who underwent cesarean sections. Out of these patients, 1009 received Quadratus Lumborum Block (QLB), while the control groups were categorized as follows: 12 studies involved no block or a false block, 8 studies utilized a transverse abdominal plane block, and 1 study employed wound infiltration. Geographically, studies were conducted in various countries with 7 studies in China, 5 studies in Poland, 3 studies in India, 2 studies in the United Arab Emirates, and 1 study in Brazil, Denmark, and Egypt, respectively. Regarding the focus of the research, acute postoperative pain was recorded in 19 studies, while chronic postoperative pain was reported in 6 studies, as presented in Table 1.

**Table 1:** Basic characteristics of included studies.

ID	Yr	Country	Type of anesthesia	QLB			Control			Outcome indicators
				Sample size	Age	Drug Dose	Sample size	Age	Drug Dose	
Michał.Bet al^[10]^	2021	Poland	Spinal anesthesia	35	32.5 ± 5.7	0.375% ropivacaine on each side	33	32.5 ± 5.7	Pretend to receive a plane block	①③④
Marcin.Met al^[11]^	2021	Poland	Spinal anesthesia	30	28.74 ± 3.25	0.375% ropivacaine on each side	28	29.29 ± 4.55	No plane block was administered	④
Michał.B et al^[12]^	2019	Poland	Spinal anesthesia	92	32.75	0.25% bupivacaine on each side	50	32.20	No plane block was administered	③④
GeetanjalT et al^[13]^	2022	India	-	30	22.6 ± 2.12	0.375% ropivacaine on each side	30	23.2 ± 2.05	Wound infiltration with 0.375% ropivacaine	②③
Sangeeta^[14]^	2020	India	Spinal anesthesia	40	27.15 ± 4.28	0.25% bupivacaine on each side	40	27.23 ± 3.62	Transversus abdominis plane block with 0.25% bupivacaine	①②③
Kalpana.V et al^[15]^	2019	India	Spinal anesthesia	30	30.0 ± 3.0	0.2% ropivacaine on each side	30	28.0 ± 3.0	Transversus abdominis plane block with 0.2% ropivacaine	①④
Blanco et al^[16]^	2014	The United Arab Emirates	Spinal anesthesia	25	47.6 ± 12.8	0.125% bupivacaine on each side	23	46.4 ± 13.8	0.9% normal saline	①②
Hansen et al^[17]^	2019	Denmark	Spinal anesthesia	34	32.3 ± 5.7	0.375% ropivacaine on each side	34	31.2 ± 5.5	0.9% normal saline	①②
Wang et al^[18,19]^	2019	China	quadratus lumborum block in combination with general anesthesia	35	26.4 ± 4.1	0.375% ropivacaine on each side	35	26.9 ± 3.8	0.9% normal saline	①②
Cai et al^[19]^	2019	China	quadratus lumborum block in combination with general anesthesia	35	26.8 ± 3.2	0.3% ropivacaine on each side	35	27.4 ± 3.9	Transversus abdominis plane block with 0.3% ropivacaine	①②
Pang et al^[20]^	2022	China	quadratus lumborum block in combination with general anesthesia	33	27.4 ± 1.33	0.25% ropivacaine on each side	33	27.32 ± 1.26	Transversus abdominis plane block with 0.25% ropivacaine	①
Wu et al et al^[21]^	2021	China	Spinal anesthesia	60	28.6 ± 4.53	0.375% ropivacaine on each side	60	29.1 ± 4.24	0.9% normal saline	①②
Yan et al^[22]^	2018	China	Spinal anesthesia	30	26.8 ± 2.7	0.25% ropivacaine on each side	30	26.4 ± 2.6	Transversus abdominis plane block with 0.25% ropivacaine	①
Cai et al^[23]^	2020	China	quadratus lumborum block in combination with general anesthesia	231	-	0.25% ropivacaine on each side	232	-	No plane block was administered	①
Michał.B et al^[24]^	2021	Poland	Spinal anesthesia	93	32.55 ± 4.8	0.25% ropivacaine on each side	94	31.48 ± 2.8	Transversus abdominis plane block with 0.25% bupivacaine	①②
Rafael. B et al^[25]^	2016	The United Arab Emirates	Spinal anesthesia	38	-	0.125% ropivacaine on each side	38	-	Transversus abdominis plane block with 0.125% bupivacaine	②
Marcin.Met al^[26]^	2018	Poland	Spinal anesthesia	28	29.15 ± 4.5	0.375% ropivacaine on each side	30	28.74 + 3.25	0.9% normal saline	①②
Karoline.et al^[27]^	2022	Brazil	Spinal anesthesia	15	33.5 ± 6.7	0.2% bupivacaine on each side	16	31.1 ± 6.4	No plane block was administered	②
Eman. R et al^[28]^	2019	Egypt	Spinal anesthesia	30	31.1 ± 5.9	0.375% ropivacaine on each side	30	32.5 ± 6.6	0.9% normal saline	②
Wang et al^[29]^	2018	China	quadratus lumborum block in combination with general anesthesia	30	24.5 ± 4.2	0.375% ropivacaine on each side	30	25.2 ± 3.8	No plane block was administered	①②
Ashok. J et al^[30]^	2022	The United Arab Emirates	Spinal anesthesia	35	-	0.375% ropivacaine on each side	36	-	Transversus abdominis plane block with 0.375% ropivacaine	①②

① VAS within 24 h, ② Cumulative 24-h intravenous morphine consumption, ③ Incidence of rescue analgesia within 24 h, ④ Incidence of chronic pain at 3 mo post-surgery, ⑤ Incidence of chronic pain at 6 mo post-surgery.

**Figure 2.**
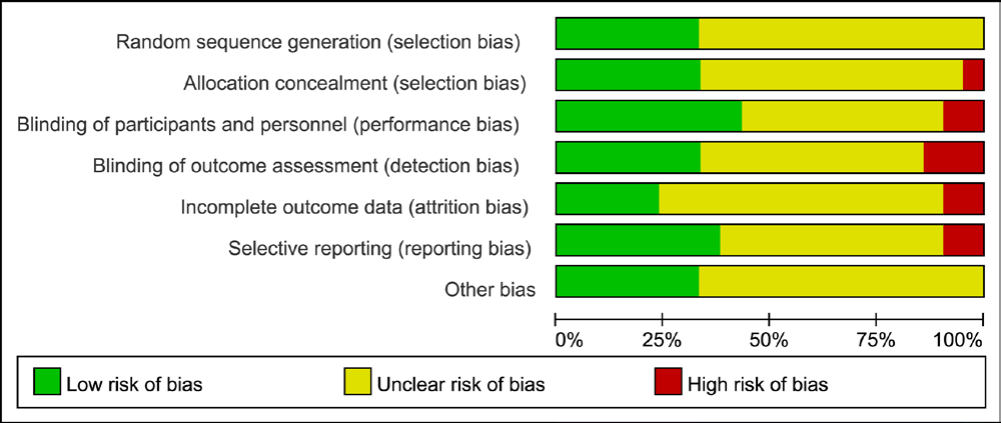
Evaluation of literature bias included in the study.

Evaluation of the quality of the included studies: According to Cochrane evaluation criteria, bias risk analysis was assessed in the included studies from 7 aspects: random sequence generation method, allocation concealment, blind method, integrity of the research results, accuracy of the result data, selectivity of the report and other bias. The evaluation results of each aspect were “low risk,” “high risk” or “unclear.” There were 14 papers with “excellent” (≥ 4 low-risk evaluation indicators) and 7 papers with “good” (≥ 2 low-risk evaluation indicators but < 4 low-risk evaluation indicators). The overall quality level of the papers was acceptable, as shown in Figure 2.

### 3.2. Meta-analysis of the analgesic effect of psoas quadratus block on acute pain after cesarean section

#### 3.2.1. VAS score 24 hours after operation.

**Figure 3.**
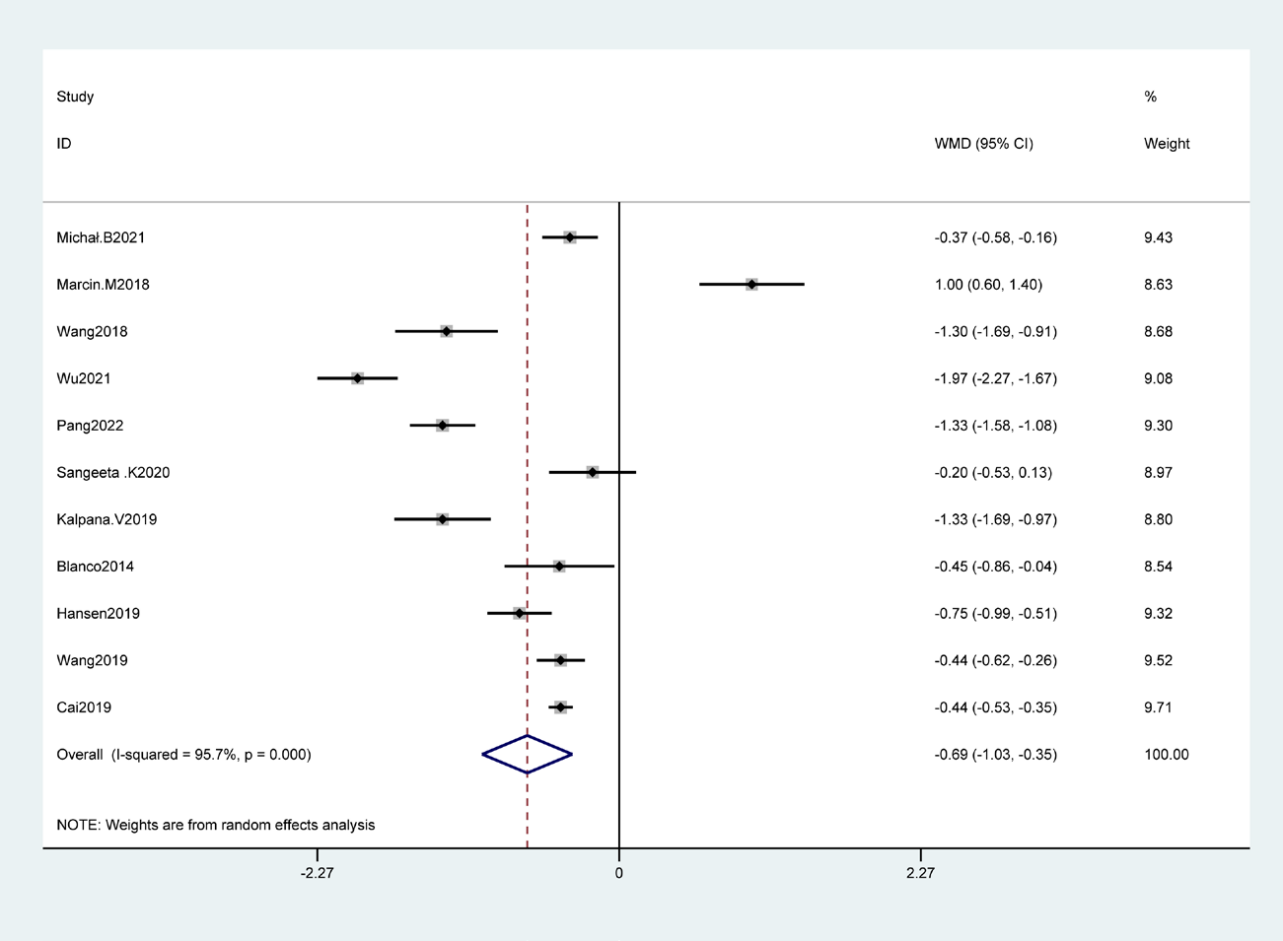
Forest plot of VAS score 24 h after operation. VAS = visual analogueanalog scale.

A total of 11 studies had reported the relationship between VAS scores of cesarean-section patients 24 hours after the operation, including 877 cases of cesarean-section patients, 443 cases in the QLB group, and 444 cases in the control group. The results showed that the QLB group could significantly reduce the VAS score 24 hours after the operation as compared with the control group (WMD = −0.69, 95% Cl: −1.03 to −0.35, *P* < .001; Heterogeneity test results: *P* < .001, I^2^ = 95.7%). There was significant heterogeneity between groups, and a random effect model was used for analysis. After subgroup analysis of the VAS score 24 hours after operation according to the type of control group and the study area, the heterogeneity was still high (I^2^ = 92.9%, I^2^ = 94.2%), as shown in Figure 3.

#### 3.2.2. Cumulative consumption of morphine for intravenous injection in 24 hours.

**Figure 4.**
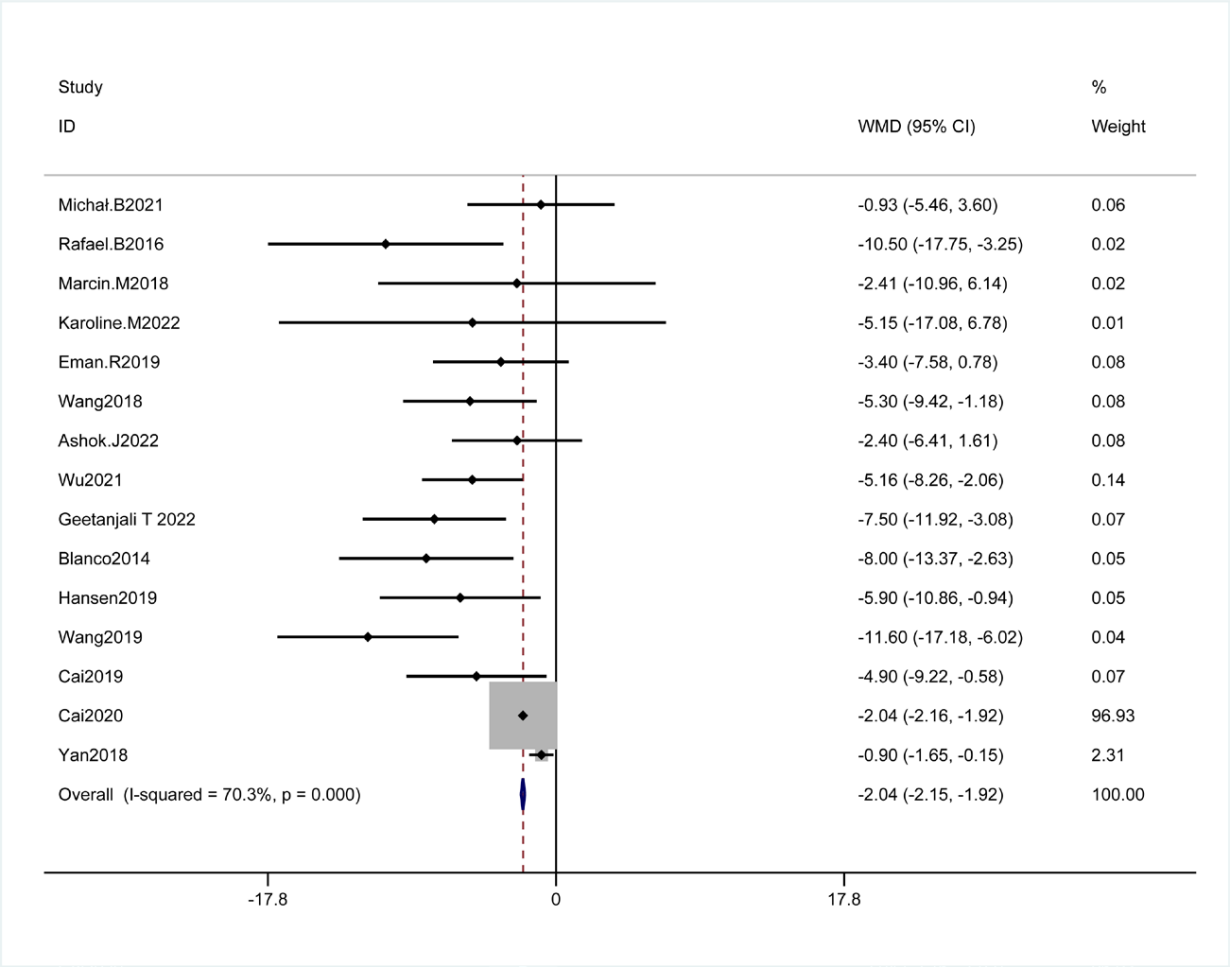
Forest plot of cumulative 24-h intravenous morphine consumption.

A total of 15 studies had reported the relationship between morphine consumption within 24 hours after cesarean section, including 1502 cesarean section patients, 749 in the QLB group, and 753 in the control group. The results showed that the QLB group could significantly reduce morphine consumption 24 hours after the operation as compared with the control group, (WMD = −2.04, 95% Cl: −2.15 ~ −1.92 *P* = .002; Heterogeneity test results: *P* < .001, I^2^ = 70.3%), the heterogeneity existed between groups, and the random effect model was conducted for analysis. After subgroup analysis of the VAS score 24 hours after operation based on the type of control group and the study area, it was found that the plane block was the source of heterogeneity in the control group (I^2^ = 62.1%), as shown in Figure 4.

### 3.3. Meta-analysis of the analgesic effect of quadratus lumborum block on chronic pain after cesarean section

#### 3.3.1. Incidence of chronic pain 3 months after the operation.

**Figure 5.**
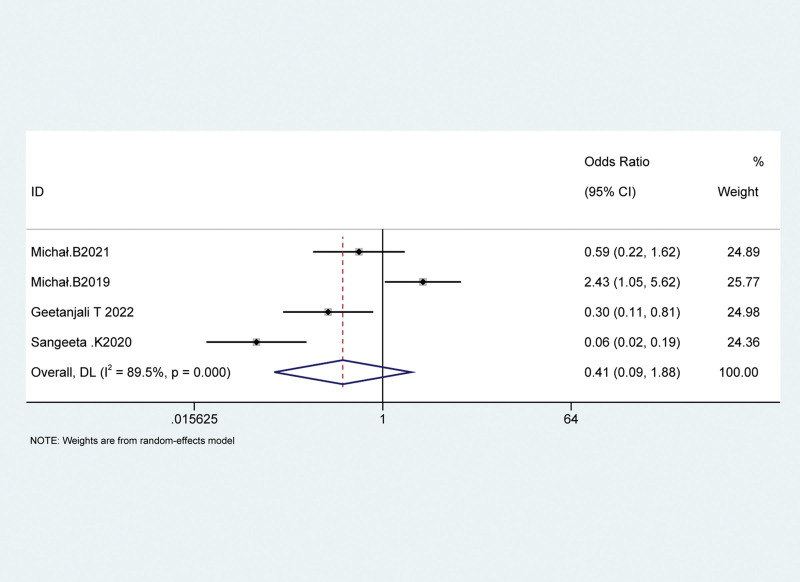
Forest plot of the incidence of chronic pain 3 mo after operation.

A total of 4 studies reported the incidence of chronic postoperative pain in patients with cesarean section 3 months after the operation, including 350 patients with cesarean section, 197 in the QLB group and 153 in the control group. The results showed that the incidence of chronic postoperative pain 3 months after surgery increased in the QLB group than the control group, but the difference was not statistically significant (OR = 0.41, 95% CI: 0.09~1.88, *P* = .253). Heterogeneity test results were *P* < .001, I^2^ = 89.5%, indicating an existing heterogeneity between the groups, and random-effects model analysis was used, as shown in Figure 5.

#### 3.3.2. Incidence of chronic pain in 6 months after operation.

**Figure 6.**
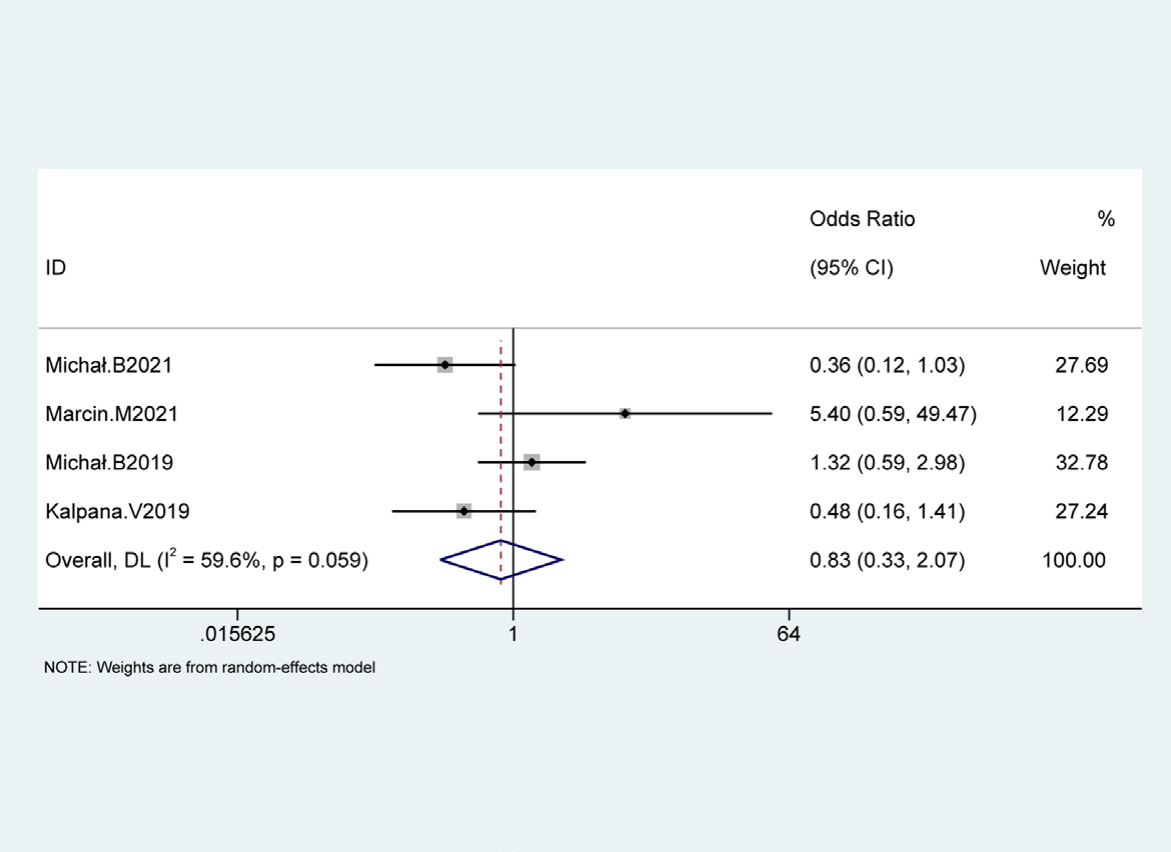
Forest plot of the incidence of chronic post-surgical pain at 6 mo post-surgery.

A total of 4 studies reported the incidence of chronic postoperative pain in patients with cesarean section 6 months after the operation, including 328 patients with cesarean section, 187 cases in the QLB group, and 141 cases in the control group. The results showed that the incidence of chronic postoperative pain 6 months after surgery in the QLB group compared to the control group has increased, but the difference was not statistically significant (OR = 0.83, 95% CI: 0.33~2.07, *P* = .686). The results of the heterogeneity test were *P* = .059, I^2^ = 59.6%, indicating an existing heterogeneity between the groups. A random-effects model was used for analysis, as shown in Figure 6.

### 3.4. Sensitivity analysis

**Figure 7.**
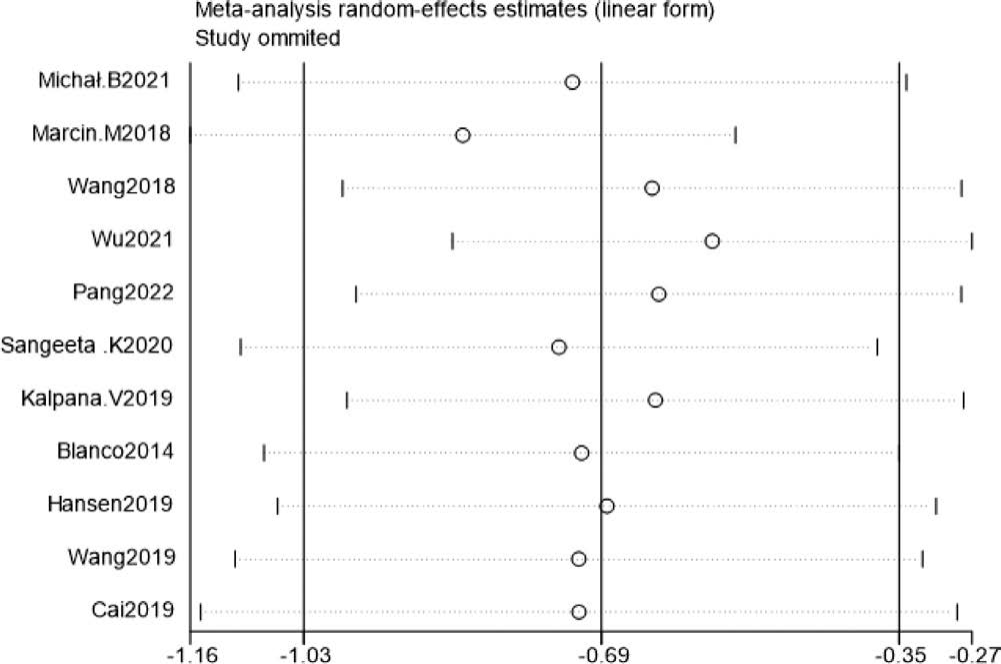
Sensitivity analysis of VAS scores within 24 h post-surgery. VAS = visual analogueanalog scale.

**Figure 8.**
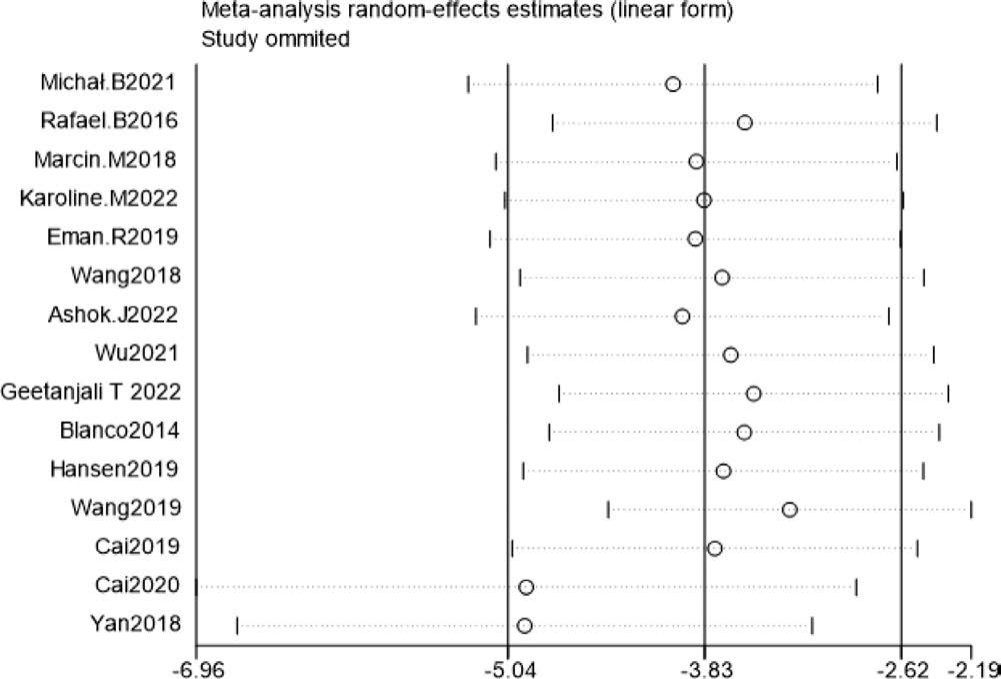
Sensitivity analysis of cumulative 24-h intravenous morphine consumption.

**Figure 9.**
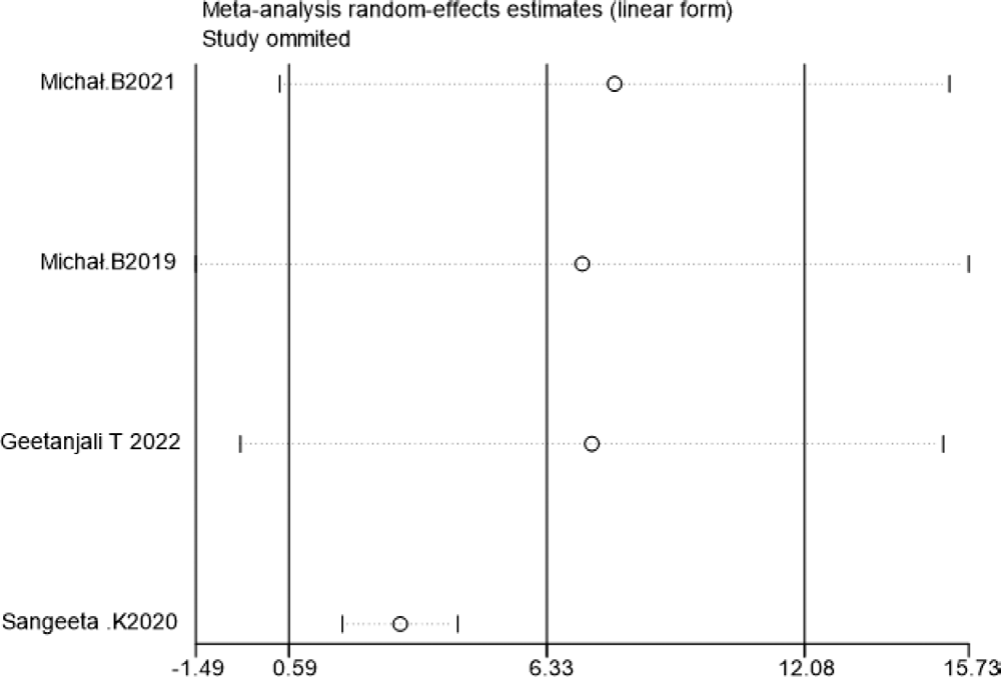
Sensitivity analysis of the incidence of chronic pain at 3 mo post-surgery.

**Figure 10.**
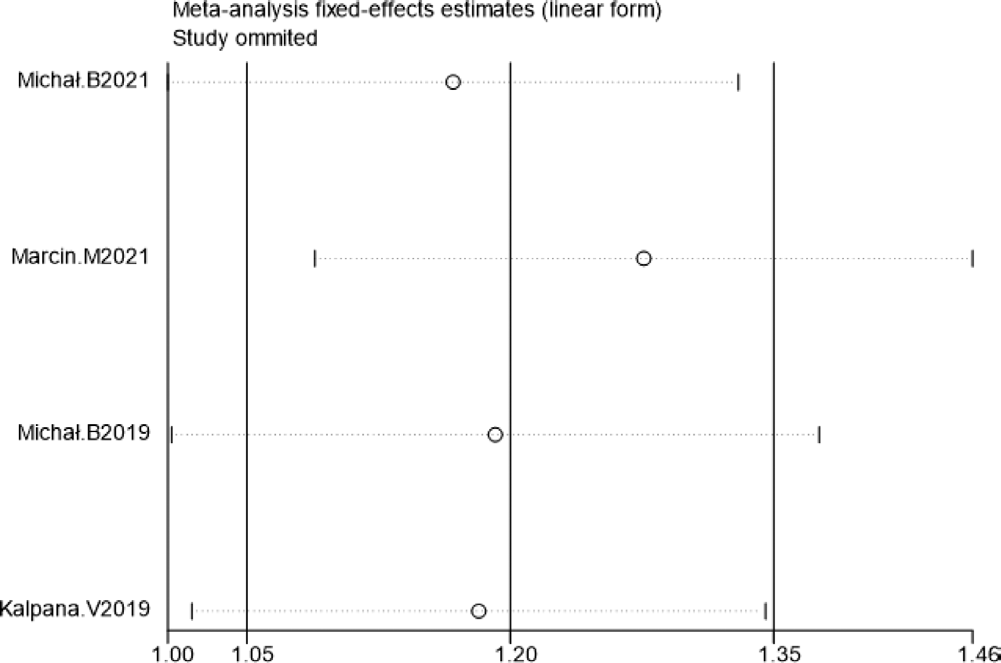
Sensitivity analysis of the incidence of chronic pain at 6 mo post-surgery.

Sensitivity analysis was performed by excluding literature one by one, and the combined effect size of each study did not change significantly, suggesting that the results of this study were relatively stable (Figs. 7–10).

### 3.5. Publication bias

**Figure 11.**
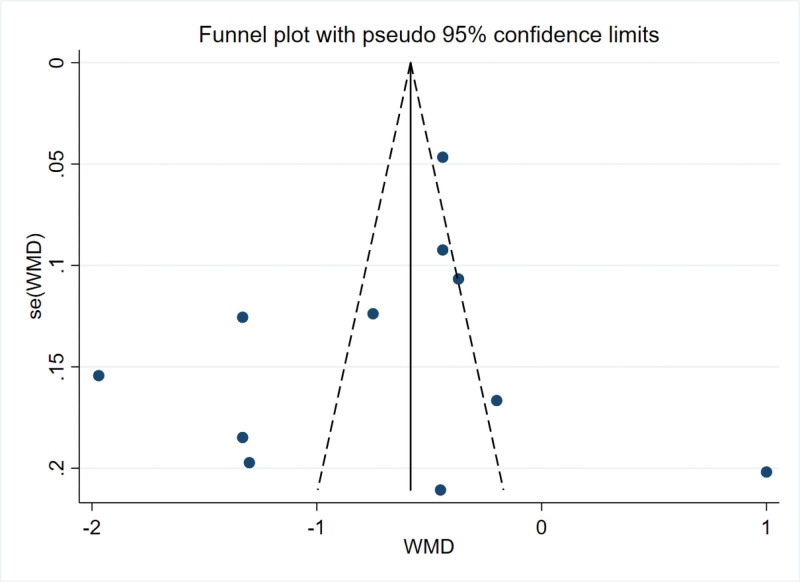
Funnel plot of VAS scores within 24 h post-surgery. VAS = visual analogueanalog scale.

**Figure 12.**
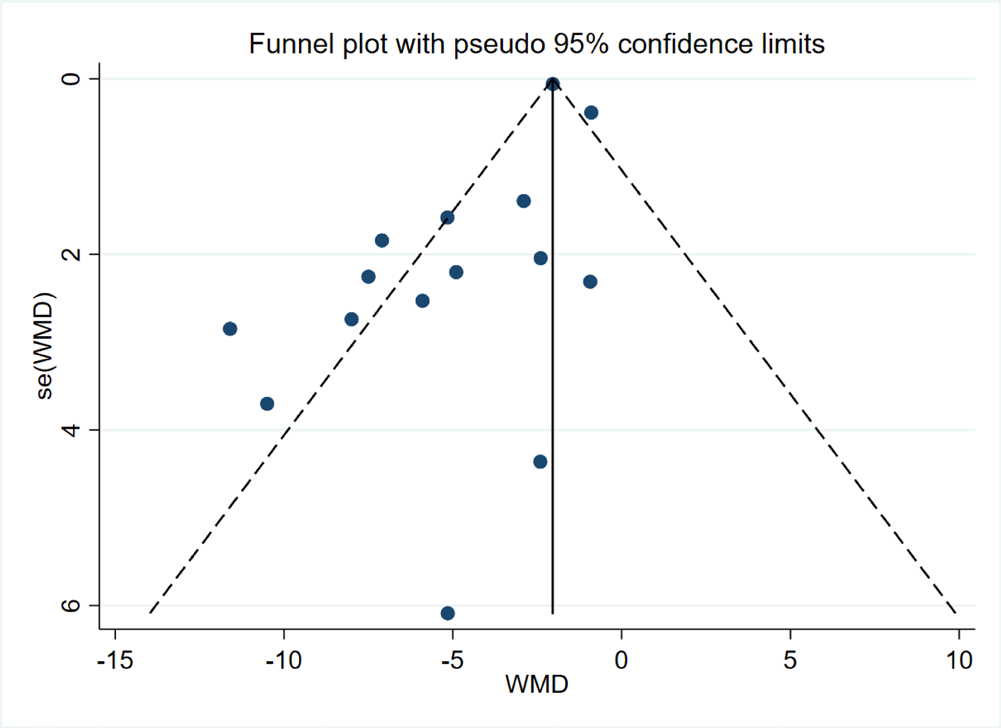
Funnel plot of cumulative 24-h intravenous morphine consumption.

The literature in this study strictly followed the inclusion and exclusion criteria. The publication bias was evaluated by the funnel map of the VAS score 24 hours after the operation and further quantified by the Egger test. The results indicated that no serious publication bias was observed (*P* = .498 > 0.05). The publication bias was evaluated by funnel chart for the cumulative 24 hours of intravenous morphine consumption after operation, and further quantified by Egger test. The results showed that there was publication bias (*P* = .016 < 0.05). Considering the relatively small number of included studies, a funnel plot analysis was not conducted for the chronic pain index. The main findings in this analysis are relatively reliable (Figs. 11 and 12).

## 4. Discussion

This meta-analysis included 21 RCTs to evaluate the analgesic effect of QLB on acute and chronic pain after cesarean section. The findings were shown as follows: In terms of QLB analgesic effect on acute pain post-cesarean section, it can reduce the VAS score and the cumulative 24-hour intravenous morphine consumption as compared to the control group. In terms of the analgesic effect of QLB on chronic pain after cesarean section, the incidence of chronic pain at 3 months and 6 months post-operation were higher when compared with the control group.

In recent years, regional blocks, as a key component of multimodal analgesia, have been increasingly used in postoperative analgesia after cesarean sections.^[31,32]^ QLB is the injection of local anesthetic into the lumbar square muscle and its surrounding fascia can spread into the interspace adjacent to the thoracic vertebrae, closer to the nerve axons and sympathetic trunks, providing satisfactory postoperative analgesia for abdominal surgery, which can provide satisfactory postoperative analgesia for abdominal surgery.^[25]^ The key to QLB block analgesia lies in the thoracolumbar fascia (TLF), a complex tubular structure made of connective tissue.^[33]^ This high block level (T7-L1) provides postoperative analgesia for both upper and lower abdominal surgery. Local anesthetics can diffuse to the paravertebral space through TLF, resulting in indirect paravertebral block, thus providing pain relief for both visceral and abdominal incision pain. Currently, there are 4 clinical approaches to QLB, namely external, posterior, anterior, and intramuscular block, and although some experts have suggested that external QLB may be a variant of TAPB, which is less effective in providing analgesia, there is no evidence from clinical trials. Kang et al demonstrated that the combination of posterior and anterior approaches can provide more satisfactory postoperative analgesia for cesarean section patients than posterior and anterior block alone. The mechanism of quadratus lumbrum block is unclear, and there may be inconsistent anesthesia platforms among studies, which needs more basic and clinical studies to prove.

The current study showed similar findings with previous studies in that QLB block, which was sufficiently analgesic for pain management after abdominal surgery.^[34]^ Meta-analyses conducted by Zhigang Zhao et al have confirmed the analgesic effects of QLB block on acute pain after cesarean section.^[35]^ Liu Jian et al conducted a meta-analysis of 10 RCTs, comparing the effectiveness and safety of QLB, indicating that QLB can reduce morphine consumption post-cesarean section—findings remained consistent with our study.^[36]^ Yang Xiaoli et al found that, through a meta-analysis of 7 RCTs, QLB significantly lowered VAS scores 24 hours after operation compared to transverse abdominal plane block. However, this was in contrast to the results of this study, which may be because this study control group included a placebo group, transverse abdominal plane block, and wound infiltration.^[37]^

The consequences of chronic pain after a cesarean section can have serious implications, affecting patient emotional well-being, social interactions, and overall medical outcomes. At present, there are a lack of meta-analysis studies that have examined the differences of QLB block in the treatment of chronic postoperative pain. This study evaluated the analgesic effect of QLB on chronic pain after cesarean section in patients receiving QLB. No differences were found in this study for the incidence of chronic pain between the QLB and control groups. This finding remained consistent with the results of a prospective observational study by Michał Borys et al^[12]^ In addition, Karmakar et al did not observe a reduction in chronic pain in a similar patient group in their study,^[38]^ which may be due to the lack of consistency in measurement and reporting in individual studies, the small sample size, and the potential for selection bias due to a lack of randomization.

## 5. Strength and limitation

There are still some limitations in this systematic review. Firstly, although we used uniform criteria to screen the literature, there was still some heterogeneity among the studies, which may be related to the different access routes for QLB, different drugs used for intrathecal anesthesia, cesarean section surgical procedure, concentration and amount of nerve block medication, and ethnicity among the studies. In addition, considering the diversity of lumbar-square muscle block access routes and types of anesthetic drug concentrations and doses, as well as the heterogeneity of studies, further studies on lumbar-square muscle block access routes and the use of anesthetic drugs will be needed to confirm our findings. Thirdly, the pain index was evaluated by a subjective scale, and future studies can use more objective data analysis such as sensors to compare the effect of the QLB group in postoperative pain management. Our meta-analyses including RCTs could provide clinical evidence to help inform decision-making for patients, physicians, and policymakers.

## 6. Conclusion

By incorporating including RCTs in this meta-analysis, this study discovered that the use of quadratus lumborum tissue for post-cesarean section analgesia, particularly for postoperative acute pain relief, showed a notable effect, which resulted in a reduction in the VAS score and morphine consumption within 24 hours after surgery. Further studies with larger sample sizes and higher quality RCTs are needed to elucidate the analgesic effect of quadratus lumborum tissue on postoperative chronic pain.

## Author contributions

**Conceptualization:** Honghong Du, Xiuqin Luo.

**Data curation:** Honghong Du, Xiuqin Luo, Min Chen, Siren Shi, Jianyong Zhao.

**Formal analysis:** Min Chen, Siren Shi.

**Project administration:** Jianyong Zhao.

**Writing – original draft:** Honghong Du, Xiuqin Luo, Min Chen, Siren Shi, Jianyong Zhao.

**Writing – review & editing:** Honghong Du, Xiuqin Luo, Min Chen, Siren Shi, Jianyong Zhao.
